# Reduced glucocorticoid production rate, decreased 5α-reductase activity and adipose tissue insulin sensitization following weight loss

**DOI:** 10.2337/db08-0094

**Published:** 2008-03-13

**Authors:** Jeremy W Tomlinson, Joanne Finney, Beverly A Hughes, Susan V Hughes, Paul M Stewart

**Affiliations:** 1 Division of Medical Sciences, Institute of Biomedical Research, University of Birmingham, Queen Elizabeth Hospital, Edgbaston, Birmingham, UK. B15 2TT; 2 Wellcome Trust Clinical Research Facility, University Hospitals Birmingham NHS Foundation Trust, Birmingham, UK. B15 2TH

**Keywords:** Obesity, 5α-reductase, 11β-hydroxysteroid dehydrogenase, cortisol, weight loss

## Abstract

**Objective:**

The epidemic of obesity, insulin resistance and type 2 diabetes has heightened the need to understand mechanisms that contribute to their pathogenesis. Increased endogenous glucocorticoid (GC) production has been implicated based upon parallels with Cushing’s syndrome. We have assessed the impact of weight loss upon GC secretion and metabolism (notably 11β-hydroxysteroid dehydrogenase type 1 (11β-HSD1) and 5α-reductase (5αR) activity) and insulin sensitivity.

**Research Design and Methods:**

20 obese volunteers were investigated before and after weight loss. Patients underwent hyperinsulinemic euglycemic clamps with simultaneous adipose microdialysis and oral cortisone acetate administration. Changes in GC secretion and metabolism were assessed using 24h urine collections.

**Results:**

Before weight loss, fat mass correlated with GC secretion rate (total fat, R=0.46, p<0.05; trunk fat, R=0.52, p<0.05); however, GC secretion rate was inversely related to insulin sensitivity (R=-0.51, p<0.05). Hyperinsulinemia failed to suppress adipose tissue interstitial fluid glycerol release (180±50 (basal) vs. 153±10μmol (steady state), p=ns). After oral cortisone (25mg), cortisol concentrations within adipose interstitial fluid increased (4.3±1.1 vs. 14.2±2.6nmol/L, p<0.01), but glycerol concentrations didn’t change. Following weight loss, insulin sensitivity increased. Consistent with insulin sensitization, adipose tissue interstitial fluid glycerol concentrations fell under hyperinsulinemic conditions (186±16 vs. 117±9μmol, p<0.05). GC secretion decreased (11751±1520 vs. 7464±937μg/24h, p<0.05) as did 5αR activity (5αTHF/THF ratio 1.41±0.16 vs. 1.12±0.17, p<0.005).

**Conclusions:**

Obesity is associated with insulin resistance within adipose tissue and increased cortisol secretion rates, both are reversed with weight loss. Reduced 5αR activity following weight loss may decrease hypothalamo-pituitary-adrenal (HPA) axis activation and reduce GC metabolite production.

The global epidemic of obesity continues to progress at an alarming rate in both adults and children (1). The health consequences of obesity are well described with significant increases in both mortality and morbidity (2). The risks of type 2 diabetes mellitus (T2DM) and insulin resistance associated with obesity are perhaps the most alarming; once body mass index (BMI) reaches 35kg/m^2^ the risk of developing T2DM increases by 42-fold in men and in women 92-fold (3;4).

Parallels with patients with glucocorticoid (GC) excess, Cushing’s syndrome have highlighted the potential role that endogenous GCs may play upon insulin sensitivity and obesity. However, obesity and insulin resistance are not states of ‘sub-clinical’ GC excess; circulating cortisol levels are either normal or even slightly reduced (5). Although activation of the HPA axis is well described, this has been attributed to changes in body composition (6;7) and importantly, the relationship with insulin sensitivity *per se* has not been explored. Cortisol availability to bind and activate the GC receptor (GR) is modified by a series of enzymes through the concept of pre-receptor hormone metabolism. 11β-hydroxysteroid dehydrogenase type 1 (11β-HSD1) is highly expressed in liver and adipose tissue (8) and its predominant activity is to convert the inactive GC, cortisone to active cortisol and thereby locally amplify the action of GCs. In contrast, the A-ring reductases (5α- and 5β-reductase, 5αR and 5βR) inactivate cortisol (in conjunction with 3α-hydroxysteroid dehydrogenase) to its tetrahydro-metabolites (5α-tetrahydrocortisol, 5αTHF and tetrahydrocortisone, THE). The activities of both these enzyme systems can limit GC availability and also impact upon HPA axis activation, such that inhibition of 11β-HSD1 (decreasing local cortisol generation) and activation of A-ring reductases (enhancing cortisol inactivation) may drive the HPA axis.

Whilst the health consequences of obesity are severe, there is clear evidence as to the benefits of significant weight loss. A reduction of 10kg in weight is associated with a 20% reduction in total mortality (with a specific 30% reduction in diabetes related deaths), a 10mmHg reduction in systolic blood pressure and a 50% decrease in the absolute risk of development of T2DM (9). Moreover, recent data have now confirmed the dramatic improvement in morbidity and mortality following bariatric surgery which achieves sustained weight loss of between 14 and 25% (10;11). Weight loss arises when there is a net calorie deficit and energy expenditure exceeds intake. However, the precise molecular mechanisms that convey the health benefits are largely unknown and it is possible that changes in GC secretion and / or metabolism may play a role. Previously, we have shown that weight loss was associated with adipocyte specific increases in 11β-HSD1 mRNA expression, but no change in global 11β-HSD1 activity (12). In light of recent studies that have highlighted the role of selective 11β-HSD1 inhibitors as agents that cause insulin sensitization (13–16), we have conducted a detailed clinical study that has examined the relationship between insulin sensitivity (both globally and locally within adipose tissue) and GC secretion and metabolism before and after significant weight loss. The underlying hypothesis is that changes in GC secretion and metabolism, may be a crucial determinant of global and tissue specific insulin sensitivity.

## Research Design and Methods

The study was approved by South Birmingham Local Research Ethics Committee and all subjects gave their informed, written consent. 20 obese volunteers (10 male, 10 female, mean age±s.d. 42±3years, mean BMI 36.6±1.0kg/m^2^) were recruited following local advertisement and underwent the clinical protocol described below. Patients had no significant past medical history and none had received glucocorticoid therapy. All patients had normal blood counts, fasting glucose, renal, liver and thyroid function. Patients with diabetes mellitus were excluded from the study.

### Clinical protocol

#### Day 1

Subjects were investigated in the fasting state. Fasting blood samples were drawn at 09.00h for measurement of total cholesterol, triglycerides, cortisol, cortisone, glucose and insulin. Measurements of BMI, waist circumference (measured supine, at the level of the umbilicus) and hip circumference (at the level of the greater trochanter) and blood pressure (average of three readings, measured supine after 10 minutes rest using Dynamap®, Critikon, Tampa, USA) were also taken. In addition, all patients performed a 24-hour urine collection for corticosteroid metabolite analysis using gas chromatography / mass spectrometry as previously described (17).

Body composition analysis was performed using dual energy X-ray absorptiometry (DEXA) with a total body scanner (QDR 45OO, Hologic inc., Bedford, MA). Coefficients of variation for multiple scans were less than 3%. Regional fat mass (trunk and leg) was analyzed as previously described (12).

#### Day 2

Subjects were again investigated in the fasted state, water being available *ad libitum* and underwent a hyperinsulinemic (40 mU/m^2^.min) euglycemic clamp for 240 min with simultaneous adipose tissue microdialysis.

Subjects were asked to abstain from caffeine, alcohol and heavy exercise for the preceding 24h. At 08.00h an adipose tissue microdialysis catheter (CMA60, CMA microdialysis, Stockholm, Sweden) was inserted into subcutaneous adipose tissue under local anaesthetic (2mL of 1% lidocaine) 10cm lateral to the umbilicus. The CMA60 catheters are 0.5mm in diameter and 50mm in length, the distal 30mm consisting of a semi-permeable membrane with a 20kDa cut-off. Following a flush sequence (15μL/min for 5 min), microdialysis was performed at a rate of 0.5μL/min and continued for 30 minutes prior to sample collection as described previously (18).

**Figure 1:**
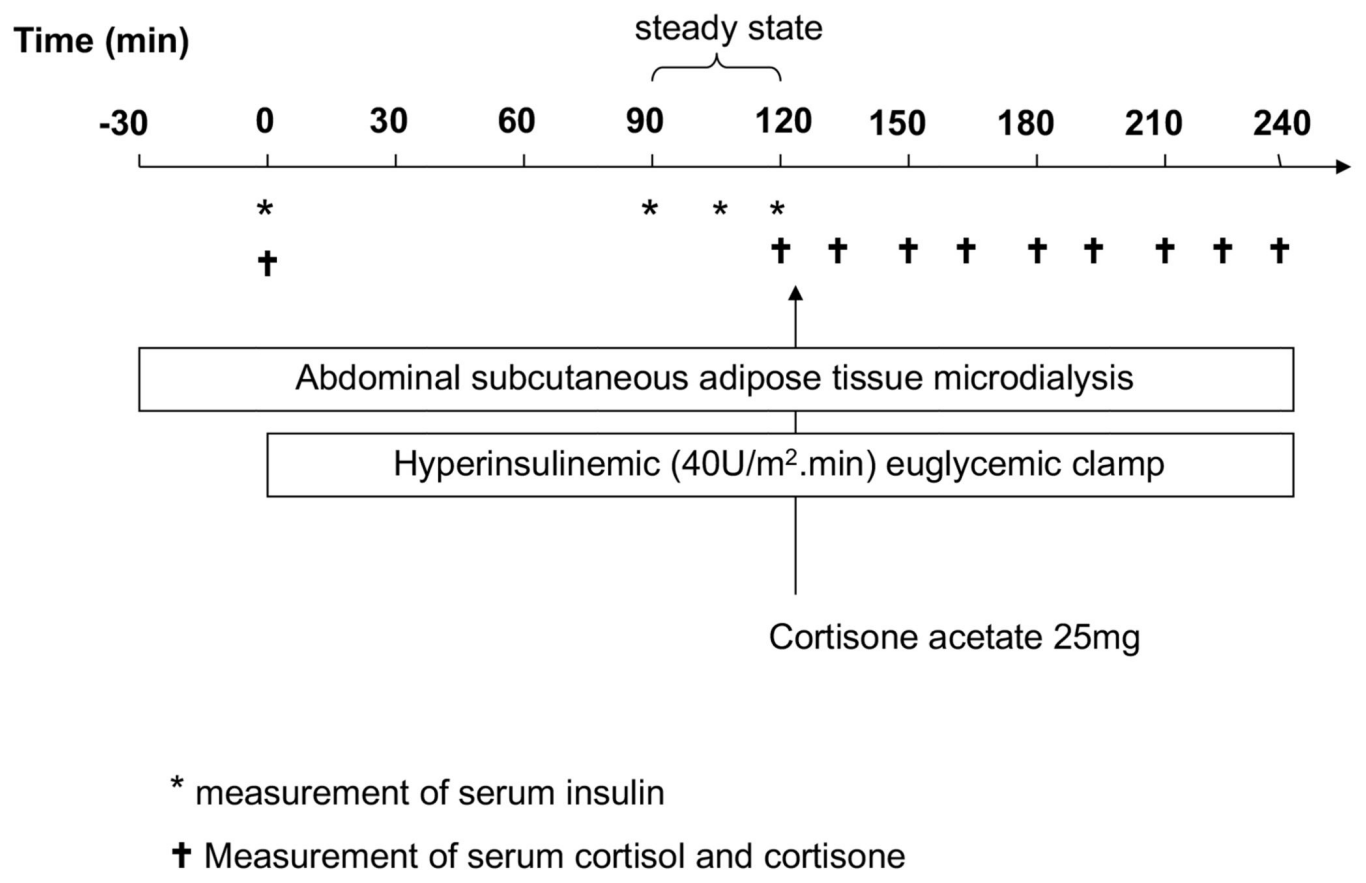
A schematic representation of the hyperinsulinemic euglycemic clamp protocol with cortisone administration and simultaneous adipose tissue microdialysis. Timing of samples taken for measurement of serum insulin (*) and cortisol and cortisone (†) are shown. Throughout the clamp, blood samples were also taken every 5 minutes for measurement of blood glucose.

The first sample was collected prior to the commencement of the hyperinsulinemic, euglycemic clamp, and sample collection continued for its entire duration (240min). Cannulae were inserted into an antecubital vein (glucose/insulin infusion) and into a contra-lateral dorsal hand vein for repeated sampling. The hand was placed in a hot-air box maintained at 50-55°C to arterialise the blood. After resting for 15min, a baseline blood sample was obtained for measurement of fasting blood glucose, insulin, cortisol and cortisone. Intravenous infusion of human soluble insulin (Actrapid, Novo Nordisk, Copenhagen, Denmark) was then started initially with a decreasing loading dose (over 10min) and then continuing for the remainder of the clamp (10-240min) at a rate of 40U/m^2^.min. 20% dextrose was infused (starting at minute 4) at a variable rate to maintain blood glucose concentrations at the fasting level. Blood glucose monitoring was performed every 5 min and measured on a bed-side blood glucose analyser (YSI incorporated, Yellow Springs, Ohio, USA ). Steady state was taken as 90-120min and during this time period 3 serum samples were taken for measurement of insulin concentrations. Glucose utilization during the steady-state period was expressed per kg body weight (M-Value) and insulin sensitivity (M/I-value) defined as the M-value divided by the mean insulin concentration as previously described (19). At t=120, further blood samples were taken for measurement of serum cortisol and cortisone. Cortisone acetate 25mg was then administered orally and subsequently additional serum samples taken every 15min for a further 2h (120-240min) for measurement of cortisol and cortisone as an index of 11β-HSD1 activity. During the 120-240min period the insulin infusion continued with 5-minute blood sampling for blood glucose concentrations. The dextrose infusion rate continued to be adjusted in order to maintain euglycaemia. A schematic representation of this protocol is shown in figure 1.

### Very Low Calorie Diet

After all investigations outlined in the clinical protocol had been completed, patients were entered on to a weight loss program using a total meal replacement, VLCD (Lipotrim, Howard Foundation, Cambridge, UK) (12). This diet provides 425 (female) and 559 (male) kcal/day. Following significant weight loss (>10% initial body weight), all subjects returned to a normal diet, and once re-feeding had been commenced for at least 1 week, all the investigation described above were repeated. Investigations were not repeated sooner so as to avoid the confounding effect that the stress of the hypocaloric diet may have had upon the HPA axis.

### Biochemical assays

#### Serum

Blood counts, urea, creatinine and electrolytes, cholesterol, triglycerides, liver chemistry and plasma glucose were measured using standard laboratory methods (Roche Modular system, Roche Ltd, Lewes, UK). Cortisol was measured using a coat-a-count radio-immuno assay (Diagnostic Products Corporation, Los Angeles, CA) as per the manufacturers guidelines. Cortisone was assayed after extraction from serum followed by radioimmunoassay (RIA) of the extract with ^125^I-Cortisone and Sac-Cel® (IDS ltd., Tyne and Weir, UK) second antibody separation (20). The coefficient of variation for 10 consecutive assays was less than 15% for values between 50 and 100nmol/L and less than 10% for values over 100nmol/L.

#### Microdialysate

Microdialysate samples were collected in microvials and exchanged every 30 minutes. Samples were analyzed using a mobile photometric, enzyme-kinetic analyzer (CMA 600, Sweden) for glucose, pyruvate, lactate, and glycerol.

Cortisol was measured using a commercially available colourimetric competitive ELISA (R and D systems, Minneapolis, MN). The minimum detectable dose range for the assay was 30-111pg/mL with intra-assay CVs of 6-9%.

#### Urinary corticosteroid metabolites

Urinary corticosteroid metabolite analysis was performed by GC/MS as described previously (17). The sum of total cortisol metabolites (THF (tetrahydrocortisol), THE (tetrahydrocortisone), 5α-THF, α-cortolone, cortisone (E), cortisol (F), β-cortolone, β-cortol, α-cortol) provides a reflection of cortisol secretion rate. The ratio of tetrahydrometabolites of cortisol (THF + 5αTHF) to those of cortisone (THE) provides a reflection of 11β-HSD1 activity when considered with the ratio of urinary free cortisol (UFF) to cortisone (UFE) which more accurately reflects renal 11β-HSD2 activity (17). In addition, the ratios of cortols:cortolones and of (11β-hydroxy-etiocholanolone + 11β-hydroxy-androsterone)/11oxo-etiocholanolone also reflect 11β-HSD1 activity (21). The activities of 5α and 5β-reductases can be inferred from measuring the ratio of 5αTHF/THF and androsterone/etiocholanolone.

### Statistical analysis

Data are presented as mean±SE unless otherwise stated. Power calculations and cohort size requirements were calculated based upon observations from our previous studies utilizing a VLCD (12). They took into account the possibility of a high drop-out rate bearing in mind the intensity of the clinical protocol. Area under the curve (AUC) analysis was performed using the trapezoidal method. For comparison of single variables before and after weight loss, paired t-tests have been used (or non-parametric equivalents where data were not normally distributed). Where repeated samples were taken (either during an individual investigation or for comparison of the same investigation before and after weight loss) repeated measures ANOVA on Ranks was used incorporating Dunn’s test as a *post hoc* analysis. Regression analyses were performed using Pearson correlations, where more than one variable was considered, multiple linear regression analysis was used. All analysis was performed using the SigmaStat 3.1 software package (Systat Software, Inc. Point Richmond, CA).

## Results

### Baseline analysis

**Figure 2:**
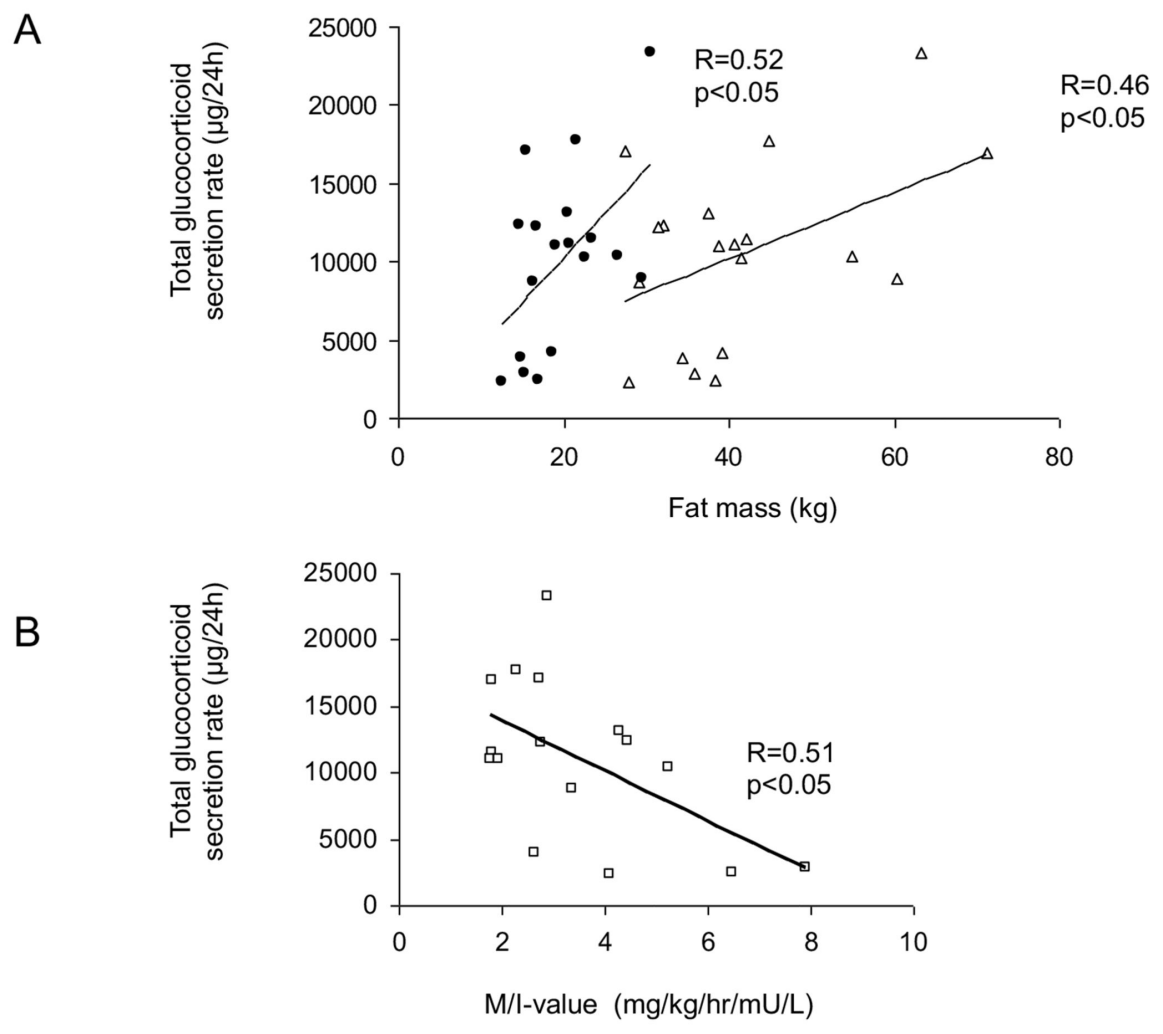
Total GC secretion rate correlates positively with trunk (closed circles) and total fat mass (open triangles) in 20 health obese individuals before weight loss (A) and inversely correlate with insulin sensitivity as measured by hyperinsulinemic euglycemic clamp (B).

Baseline characteristics of the patients are presented in table1. Prior to weight loss, total fat mass and trunk fat mass correlated with total GC secretion rate (total fat, R=0.46, p<0.05; trunk fat, R=0.52, p<0.05, figure 2A). There was no significant correlation between fat mass (total or trunk) and insulin sensitivity as measured by the M/I value (total fat, R=-0.17, p=0.52; trunk fat, R=-0.11, p=0.68). However, the M/I value was inversely related to GC secretion rate (R=-0.51, p<0.05), even following correction for fat mass (p<0.05).

Within this cohort, insulin sensitivity and fat mass (total or regional) did not correlate significantly with 11β-HSD1 activity as measured by either urinary THF+5αTHF/THE ratio or the generation of cortisol following oral cortisone.

#### Adipose tissue microdialysis

During the steady-state period (90-120min), adipose tissue interstitial fluid glucose concentration did not differ significantly from basal glucose values (2.2±0.2 vs. 2.7±0.2mmol/L, p=ns) confirming that in addition to successful clamping of blood glucose (4.9±0.2 (fasting) vs. 4.8±0.1mmol (steady state), p=ns), adipose tissue interstitial fluid glucose concentrations had also been clamped. Consistent with insulin promoting glucose utilization, interstitial fluid pyruvate (38.1±5.8 (basal) vs. 77.5±5.2μmol/L (steady state), p<0.05) and lactate (0.68±0.1 (basal) vs. 2.1±0.2mmol/L (steady state), p<0.05) increased significantly. Basal glycerol release did not correlate with insulin sensitivity, and consistent with underlying insulin resistance in this obese cohort, hyperinsulinemia failed to suppress glycerol release (180±50 (basal) vs. 153±10μmol/L (steady state), p=ns).

Under hyperinsulinemic conditions, oral cortisone acetate administration did not have any impact upon interstitial fluid glucose, lactate, pyruvate or glycerol concentrations (data not shown).

Consistent with previous observations (18) cortisol was detectable in adipose tissue interstitial fluid and concentrations were not altered by hyperinsulinemia (4.3±1.1 (basal) vs. 5.4±1.0nmol/L (steady state, 90-120min), p=ns). Following oral cortisone acetate administration, interstitial fluid cortisone concentrations increased significantly (14.2±2.6nmol/L, 210-240min, p<0.005 vs. 90-120min).

### Impact of weight loss

Of the 20 patients who were recruited and enrolled into the study, 14 (7 men, 7 women) completed the full investigative protocol using the VLCD for 10 (7–15) weeks (median and range). 6 volunteers were unable to tolerate the VLCD and were not reinvestigated

#### Anthropometry, fasting biochemistry and blood pressure

Following the VLCD, mean BMI fell from 36.6±1.3 to 31.4±1.0kg/m^2^ (n=14, p<0.0001). We observed significant reductions in waist circumference, total and trunk fat mass, lean mass and percentage fat (table 2). However, there was little change in fat distribution as waist/hip ratio and trunk/limb fat mass ratio on DEXA scanning remained unchanged (table 2).

Consistent with a decrease in muscle mass, serum creatinine decreased significantly (97±2 vs. 91±2μmol/L, p<0.05). Total cholesterol decreased with no significant change in triglyceride concentrations. Complete data are presented in table 3. In addition, systolic blood pressure fell significantly (129±3 vs. 119±3mmHg, p<0.005), without alteration in diastolic blood pressure (74±3 vs. 70±2mmHg, p=0.26).

#### Insulin sensitivity

Whilst fasting plasma glucose remained unchanged (5.2±0.2 vs. 4.8 ±0.1mmol/L, p=0.16), fasting insulin fell significantly (15.0±1.9 vs. 8.3±1.9mU/L, p<0.01). Glucose disposal as reflected by the M-value increased (3.2±0.3 vs. 5.1±0.7mgUkg/min, p<0.005) as did insulin sensitivity (M/I-value) (3.7±0.5 vs. 7.0±1.1mg/kg/min/mU/L, p<0.005). Calculated measures of insulin sensitivity (HOMA) also increased (table 3).

#### Cortisol secretion and metabolism

Following weight loss, total GC secretion decreased (11751±1520 vs. 7464±937μg/24h, p<0.05). Decreases in absolute levels of 5α-reduced metabolites were more marked compared to 5β-reduced metabolites (table 4), consistent with decreased 5αR activity (Androsterone/Etiocholanolone 1.65±0.23 vs. 1.2±0.19, p=0.002; 5αTHF/THF 1.41±0.16 vs. 1.12±0.17, p=0.004). Urinary steroid metabolite ratios that reflect 11β-HSD1 activity (THF+5αTHF/THE and cortols/cortolones) did not change significantly with the exception of the 11OH-androsterone + 11OH-etiocholanolone / 11oxo-etiocholanolone ratio which decreased (2.20±0.23 vs. 1.65±0.22, p=0.01) (table 4).

Consistent with our previous observations (12), cortisol generation from oral cortisone within serum did not change following weight loss (AUC 938±179 (before) vs. 854±97nmol/L.h (after), p=0.44) (figure 4A).

#### Adipose tissue microdialysis

**Figure 3:**
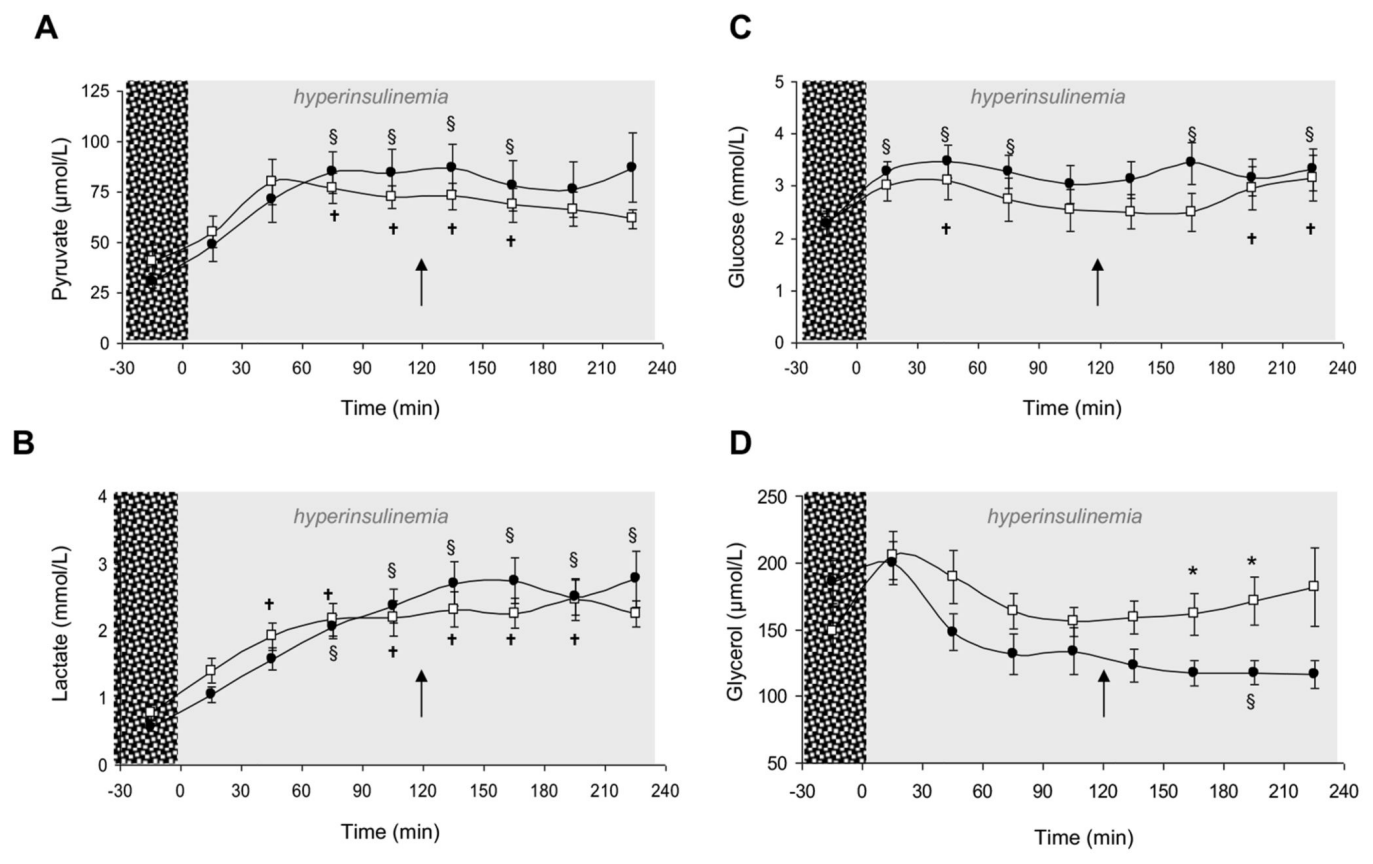
Adipose tissue microdialysate analysis in 14 obese individuals before (open squares) and after weight loss (closed circles), under basal and hyperinsulinemic conditions. Hyperinsulinemia increased interstitial fluid pyruvate (A) and lactate (B) concentrations equally before and after weight loss. Glucose concentrations were unaltered consistent with successful clamping of blood glucose levels and were also similar before and after weight loss. Glycerol concentrations failed to suppress prior to weight loss in keeping with adipose tissue insulin resistance, but fell significantly after weight loss († p<0.05 vs. baseline before weight loss; § p<0.05 vs. baseline after weight loss; * p<0.05 before vs. after weight loss. Arrow denotes administration of cortisone acetate 25mg orally).

After weight loss, hyperinsulinemia increased pyruvate and lactate concentrations in adipose tissue interstitial fluid (as observed before weight loss) although values before and after weight loss were not different (figure 3A and B). Similarly, there were no significant differences in glucose concentrations (figure 3C).

In contrast to the results before weight loss, glycerol concentrations fell under hyperinsulinaemic conditions, consistent with repression of lipolysis (186±16 (basal) vs. 117±9μmol/L (180-210min), p<0.05). Furthermore, glycerol levels were significantly reduced compared with before weight loss suggesting insulin sensitization (162±16 vs. 117±10μmol/L, p<0.05 and 171±18 vs. 117±9μmol/L, p<0.05) (figure 3D).

**Figure 4:**
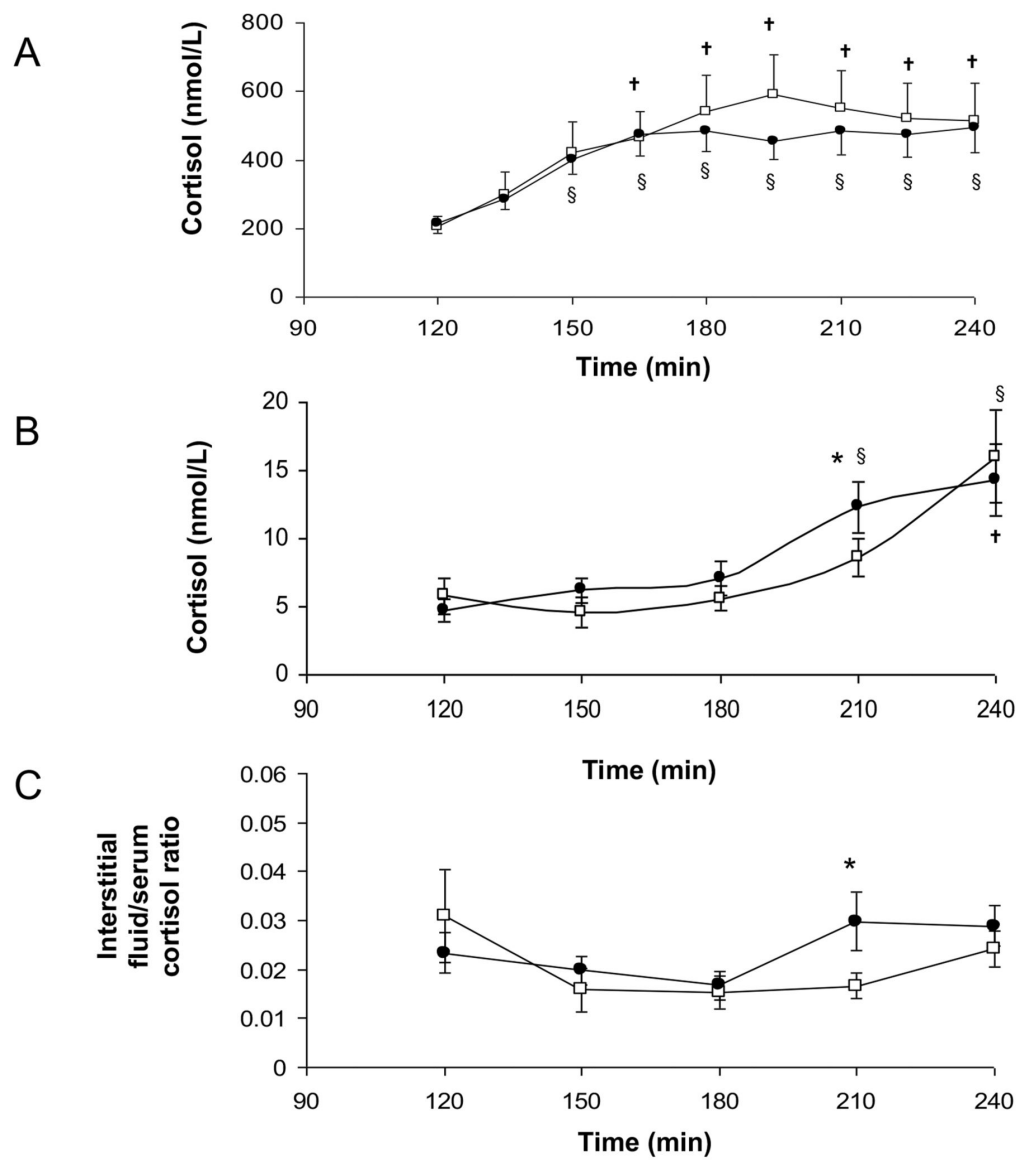
Serum (A) and adipose tissue interstitial fluid (B) cortisol generation, and the ratio of interstitial fluid cortisol to serum cortisol (C) following oral cortisone acetate (25mg) administration in 14 obese individuals before (open squares) and after weight loss (closed circles) († p<0.05 vs. baseline before weight loss; § p<0.05 vs. baseline after weight loss).

Cortisol concentrations in adipose tissue interstitial fluid at the t=180-210min time point, were higher after weight loss (8.6±1.4 (before) vs. 12.3±1.9nmol/L (after), p=0.03) (figure 4B), however total cortisol production as measured by area under the curve analysis did not differ (AUC 12.8±1.9 (before) vs. 13.8±2.3nmol/L.h (after), p=0.23) (figure 4B). Before and after weight loss, immediately after the administration of oral cortisone acetate, the ratio of interstitial fluid:serum cortisol decreased (figure 4C) suggesting a more rapid appearance of cortisol in serum rather than interstitial fluid. After weight loss, there was a significant increase in interstitial fluid cortisol availability compared to serum (p<0.05 vs. before weight loss) (figure 4C) consistent with enhanced local generation of cortisol.

## Discussion

We have characterized GC secretion and metabolism and insulin sensitivity in a cohort of obese individuals before and after weight loss. Total GC secretion rate was related to both regional and total fat mass and inversely correlated with insulin sensitivity. Interestingly, in this cohort, there was no relationship between fat mass (or distribution) and insulin sensitivity. Prior to weight loss, adipose tissue was insulin resistant with failure of insulin to suppress lipolysis. Following weight loss, we observed marked increases in insulin sensitivity, both globally and also within adipose tissue as evidenced by insulin mediated suppression of glycerol release. Furthermore, we observed a decrease in total GC secretion rate and a specific reduction in 5αR activity.

The role of endogenous GC production and metabolism has been implicated in the pathogenesis of obesity and insulin resistance. Much of this work has cited phenotypic similarities between patients with Cushing’s syndrome and those with simple obesity. Activation of the HPA axis and hypersensitivity of the axis to stimulation and suppression have been described previously in obesity (6;7). However, the independent relationship between GC secretion and insulin sensitivity is a novel and interesting finding; we suggest that the increased adrenal GC output could have an impact upon insulin sensitivity and glucose utilization in liver, muscle and perhaps adipose tissue.

The majority of GC metabolism occurs within the liver. Over recent years there has been a focus on the role of 11β-HSD1 as a local amplifier of cortisol action in the pathogenesis of obesity and insulin resistance (22). Moreover, inhibitors of 11β-HSD1 have shown considerable potential in rodents (13–16) and primates (23) as insulin sensitizers and as agents that may aid weight loss. We were unable to show significant changes in 11β-HSD1 activity in either serum or urine in agreement with our previous observations (12). However, our data do suggest increased cortisol availability within adipose tissue interstitial fluid following weight loss. Previously, we have shown increased adipocyte (but not whole adipose tissue) expression of 11β-HSD1 following weight loss (12) and this may be responsible for the modest changes that we observed, although we have not evaluated adipocyte specific gene expression in this study. Insulin resistance in obesity specifically within adipose tissue has long been postulated, but not demonstrated. Insulin is a potent suppressor of adipose tissue lipolysis and the lack of effect of hyperinsulinemia upon adipose tissue interstitial glycerol levels before weight loss is suggestive of insulin resistance. These observations contrast with those after weight loss when hyperinsulinemia decreased glycerol concentrations consistent with adipose tissue insulin sensitization. The modest increase in cortisol following cortisone acetate suggestive of increased (and certainly not decreased) cortisol availability may be important here bearing in mind the recent description that cortisol can sensitize insulin action in subcutaneous adipose tissue (24).

GCs have potent action upon insulin signalling. Globally they induce whole body insulin resistance (25), perhaps through induction of lipolysis through hormone sensitive lipase activation (26) and subsequent free fatty acid generation. Tissue specificity of the interaction between GCs and the insulin signalling cascade is controversial and in rodents in the majority of published studies GCs appear to cause insulin resistance in adipose tissue (27;28). We have recently extended these findings to humans, demonstrating insulin resistance in muscle with insulin sensitization in subcutaneous adipose tissue (24). Data from this study endorse these observations with global decreased GC secretion associated with increased insulin sensitivity (largely reflecting GC action upon skeletal muscle) and adipose tissue insulin sensitization with increased local GC availability.

The changes in 5αR activity are striking. 5αR has 2 isoforms which only share approximately 47% sequence homology (29); the type 1 isoform (5αR1) is located on the short arm of chromosome 5, whilst 5αR2 is located on the short arm of chromosome 2. 5αR1 is expressed in skin and adipose tissue (29;30) and 5αR2 in prostate, epididymis and seminal vesicles; both isozymes are expressed in the liver (29). For cortisol metabolism, studies suggest that the most significant contributor to 5α-reduction of GC metabolites within the liver is 5αR2.

In our study 5αR activity decreased significantly following weight loss. The role of 5αR in the control of body composition and insulin sensitivity has not been investigated in detail. Studies have shown enhanced 5αR activity with obesity (31) and T2DM (32) and sexual dimorphism of expression with increased activity in males (31;33). Dietary macronutrient composition has also been implicated as a regulator of 5αR and 5βR activity; high-fat, low-carbohydrate and moderate-fat, moderate-carbohydrate diets decrease 5αR and 5βR activity (34). The effect was most pronounced in the high-fat, low-carbohydrate diet, but both diets were associated with significant weight loss (34). In rodents, treatment of obese Zücker rats with insulin sensitizers decreases 5αR1 expression in the liver (35). In patients with polycystic cystic ovary syndrome, 5αR activity correlates positively with markers of insulin resistance (36). Specifically with regards to GC metabolism, reduced 5αR activity decreases the inactivation of cortisol to its tetra-hydrometabolites and as a consequence this may relax the drive to the HPA axis and be responsible for the reduction in total GC secretion that we observed after weight loss. Interestingly the converse is probably also true in PCOS where enhanced 5αR activity may drive the HPA axis to maintain circulating cortisol levels at the expense of adrenal androgen excess (37).

This study adds further evidence as to the role of the HPA axis and GC metabolism in the pathogenesis of obesity, insulin resistance and the metabolic syndrome and has identified a potentially novel role for 5αR. In addition, we have shown adipose tissue specific changes in metabolism and insulin sensitization after weight loss. Whilst we are unable to say whether the observed changes are a cause or a consequence of the improved beneficial phenotype following weight loss, this study raises the possibility that manipulation of 5αR activity, perhaps in a tissue specific manner may have potential as a therapeutic strategy.

## Figures and Tables

**Table 1:** Baseline analysis of 20 obese individuals (mean±se)

***Anthropometric measurements***	
Weight (kg)	106.3±3.7
BMI (kg/m^2^)	36.6±1.0
Waist circumference (cm)	102±6
Waist:Hip ratio	0.92±0.02
***Body composition (DEXA)***	
Total fat mass (kg)	41.6±2.8
Total lean mass (kg)	59.2±1.8
Percentage fat (%)	39.8±1.5
Trunk fat mass (kg)	19.8±1.2
Trunk fat/limb fat ratio	1.07±0.05
***Serum biochemistry and hyperinsulinemic euglycemic clamp***	
Glucose (mmol/L)	5.1±0.2
Insulin (mU/L)	13.9±1.4
HOMA %B	151.6±10.1
HOMA %S	62.3±9.9
HOMA IR	2.0±0.2
M-value (mg/kg/min)	3.2±0.3
M/I-value (mg/kg/min/mU/L)	3.5±0.4
Cholesterol (mmol/L)	5.0±0.2
Triglycerides (mmol/L)	1.32±0.17
Cortisol (180-550nmol/L)	318±40
Cortisone (nmol/L	61±4
***Urine corticosteroid metabolites***	
Total glucocorticoid metabolites (μg/24h)	10525±1285
UFF/UFE	0.75±0.06
(THF+5αTHF)/THE	0.92±0.05
Cortols/cortolones	0.42±0.03
(11OH-androst + 11OH-etio)/11oxo-etio	2.21±0.20
5αTHF/THF	1.13±0.15
Androsterone/Etiocholanolone	1.40±0.2

**Table 2:** Anthropometric measurements and body composition data as measured by DEXA in 14 individuals before and after weight loss using a very low calorie diet.

	*Before weight loss*	*After weight loss*	*p*
***Anthropometric measurements***				
Weight (kg)	106.0±4.4	91.0±3.5	<0.0001
BMI (kg/m^2^)	36.6±1.3	31.4±1.0	<0.0001
Waist circumference (cm)	112±3	100±2	<0.0001
Waist:Hip ratio	0.91±0.02	0.89±0.02	0.25
***Body composition (DEXA)***				
Total fat mass (kg)	44.0±3.4	33.4±2.6	<0.0001
Total lean mass (kg)	59.0±2.0	55.0±2.0	<0.0001
Percentage fat (%)	41.1±1.8	36.3±1.8	<0.0001
Trunk fat mass (kg)	20.6±1.4	15.1±1.1	<0.0001
Trunk fat/limb fat ratio	1.04±0.05	1.00±0.06	0.44

**Table 3:** Biochemical characterization of 14 individuals before and after weight loss using a very low calorie diet.

	*Local reference range (where applicable)*	*Before weight loss*	*After weight loss*	*p*
Na	134-146mmol/L	141±1	141±1	0.39
K	3.4-5.2mmol/L	4.1±0.1	4.2±0.1	0.44
Urea	3.2-7.6mmol/L	4.7±0.3	4.3±0.3	0.44
Creatinine	60-126μmol/L	97±2	91±2	**0.02**
Glucose (mmol/L)		5.2±0.2	4.8±0.1	0.16
Insulin (mU/L)		15.0±1.9	8.3±1.9	**0.008**
HOMA %B		156.9±13.8	111.3±11.4	**0.005**
HOMA %S		59.7±13.5	119.7±18.6	**0.002**
HOMA IR		2.2±0.3	1.2±0.3	**0.009**
Cholesterol (mmol/L)		5.1±0.3	4.5±0.3	**0.04**
Triglycerides (mmol/L)		1.4±0.2	1.2±0.2	0.09
Cortisol (nmol/L)		349±52	302±29	0.08
Cortisone (nmol/L		58±4	66±5	0.33

**Table 4:** Urinary corticosteroid metabolite analysis performed by GC/MS on 24hr urine samples from 14 obese volunteers before and after weight loss using a very low calorie diet. (THE=tetrahydrocortisone, THF=tetrahydrocortisol, UFF=urinary free cortisol, UFE = urinary free cortisone, 11OH-androst=11hydroxyandrosterone, 11OH-etio=11hydroxyetiocholanolone, 11oxo-etio=11oxo-etiocholanolone, Fm=cortisol+THF+5αTHF+α-cortol+β-cortol, Em=cortisone+THE+α-cortolone+β- cortolone)

	*Before weight loss*	*After weight loss*	*p*
***Corticosteroid metabolites (μg/24hrs)***				
Total glucocorticoid metabolites	11751±1520	7464±937	**0.02**
Total cortisol metabolites (Fm)	5194±640	3278±439	**0.02**
Total cortisone metabolites (Em)	6661±912	4288±537	**0.02**
THE	4456±682	2877±384	**0.03**
5αTHF	2275±285	1250±214	**0.002**
THF	1878±371	1304±209	0.14
UFE	120±17	103±18	0.45
UFF	78±12	70±11	0.56
α-cortolone	1405±181	861±110	**0.009**
β-cortolone	680±82	447±61	**0.02**
α-cortol	327±41	209±32	**0.03**
β-cortol	533±70	341±65	**0.06**
***Corticosteroid metabolite ratios***				
UFF/UFE	0.66±0.04	0.70±0.05	0.25
(THF+5αTHF)/THE	0.99±0.05	0.91±0.06	0.13
Cortols/cortolones	0.43±0.03	0.41±0.03	0.40
(11OH-androst + 11OH-etio)/11oxo-etio	2.20±0.23	1.65±0.22	**0.01**
5αTHF/THF	1.41±0.16	1.12±0.17	**0.004**
Androsterone/Etiocholanolone	1.65±0.23	1.20±0.19	**0.002**
